# In-hospital, short-term and long-term adverse clinical outcomes observed in patients with type 2 diabetes mellitus vs non-diabetes mellitus following percutaneous coronary intervention: A meta-analysis including 139,774 patients

**DOI:** 10.1097/MD.0000000000014669

**Published:** 2019-02-22

**Authors:** Xiaojun Zhuo, Chuanzeng Zhang, Juan Feng, Shenyu Ouyang, Pei Niu, Zhaohui Dai

**Affiliations:** a Department of Cardiology, Affiliated Changsha Hospital of Hunan Normal University, The Fourth Hospital of Changsha, Hunan, Changsha; b State Key Laboratory of Medicinal Chemical Biology, College of Pharmacy, Nankai University, the city of Tianjin, Tianjin, PR China.

**Keywords:** adverse clinical outcomes, diabetes mellitus, percutaneous coronary intervention, stent thrombosis

## Abstract

**Background:**

Several studies have shown that patients with type 2 diabetes mellitus (T2DM) have worse clinical outcomes in comparison to patients without diabetes mellitus (DM) following Percutaneous Coronary Intervention (PCI). However, the adverse clinical outcomes were not similarly reported in all the studies. Therefore, in order to standardize this issue, a meta-analysis including 139,774 patients was carried out to compare the in-hospital, short-term (<1 year) and long-term (≥1 year) adverse clinical outcomes in patients with and without T2DM following PCI.

**Methods:**

Electronic databases including MEDLINE, EMBASE, and the Cochrane Library were searched for Randomized Controlled Trials (RCTs) and observational studies. The adverse clinical outcomes which were analyzed included mortality, myocardial infarction (MI), major adverse cardiac events (MACEs), stroke, bleeding, target vessel revascularization (TVR), target lesion revascularization (TLR), and stent thrombosis. Risk Ratios (RR) with 95% confidence intervals (CI) were used to express the pooled effect on discontinuous variables and the analysis was carried out by RevMan 5.3 software.

**Results:**

A total number of 139,774 participants were assessed. Results of this analysis showed that in-hospital mortality and MACEs were significantly higher in patients with T2DM (RR 2.57; 95% CI: 1.95–3.38; *P* = .00001) and (RR: 1.38; 95% CI: 1.10–1.73; *P* = .005) respectively. In addition, majority of the short and long-term adverse clinical outcomes were also significantly higher in the DM group as compared to the non-DM group. Stent thrombosis was significantly higher in the DM compared to the non-DM group during the short term follow-up period (RR 1.59; 95% CI: 1.16–2.18;*P* = .004). However, long-term stent thrombosis was similarly manifested.

**Conclusion:**

According to this meta-analysis including a total number of 139,774 patients, following PCI, those patients with T2DM suffered more in-hospital, short as well as long-term adverse outcomes as reported by most of the Randomized Controlled Trials and Observational studies, compared to those patients without diabetes mellitus.

## 1 Introduction

Now a days people are used to a more sedentary lifestyle, and hence, the number of patients with type 2 diabetes mellitus (T2DM) is indirectly increasing annually. Excluding the number of undiagnosed cases, more than 171 million people suffer from T2DM throughout the globe .^[1]^ T2DM is often complicated by macro-vascular conditions such as coronary artery diseases (CAD) which finally leads to acute coronary syndrome.^[2]^ Patients with T2DM and co-existing CAD are often candidates of multi-vessel diseases. Silent myocardial infarction may easily lead to sudden cardiac death in such patients.

Even if Coronary Artery Bypass Grafting (CABG) is associated with better prognosis,^[3]^ Percutaneous Coronary Intervention (PCI) is the preferred mode of treatment in many patients with T2DM.

Several studies have shown T2DM to be associated with worse in-hospital, short-term, and long-term clinical outcomes following PCI in comparison to patients without diabetes.^[4,5]^ However, different studies have reported different outcomes; that is, the reported outcomes were not always similar.

Therefore, in order to standardize this issue, a meta-analysis including 139,774 patients was carried out to compare the in-hospital, short-term (<1 year) and long-term (≥1 year) adverse clinical outcomes in patients with and without T2DM following PCI.

## 2 Methods

### 2.1 Data sources and search strategy

MEDLINE, EMBASE, and the Cochrane library were searched for Randomized Controlled Trials (RCTs) and observational studies comparing post PCI outcomes in patients with vs without T2DM by typing the words “diabetes and non-diabetes and PCI”. The word “PCI” was also replaced by its full form “percutaneous coronary intervention”. To further enhance this search, the terms “angioplasty”, “drug eluting stents” were also used. ‘Google scholar was also searched for relevant publications. All references from relevant studies were also reviewed for suitable articles. No language restriction was applied.

### 2.2 Inclusion and exclusion criteria

Studies were included if:

1. They were RCTs or observational studies comparing adverse clinical outcomes in patients with vs without T2DM following PCI;
2. They reported in-hospital follow-up, short-term follow up (<1 year), or a long-term follow-up (≥1 year).

Studies were excluded if:

1. Adverse clinical outcomes were not reported among the endpoints;
2. They were meta-analyses or case studies;
3. The control group/non-diabetic group was absent;
4. They did not include data with discontinuous variables or data which could be easily converted to discontinuous variables.

### 2.3 Outcomes and follow up

The adverse clinical outcomes which were assessed included:

1. Mortality;
2. Myocardial infarction (MI);
3. Major adverse cardiac effects (MACEs) consisting of death, MI and revascularization;
4. Stent thrombosis consisting of definite and probable stent thrombosis;
5. Stroke;
6. Bleeding consisting of any type of bleeding (minor or major);
7. Target vessel revascularization (TVR);
8. Target lesion revascularization (TLR).

Follow up time period involved an in-hospital follow-up, a short-term follow up (<1 year) and a long-term follow up (1 year or more).

*In-hospital follow-up time period*: was defined as a follow-up period during the in hospital stay following PCI. However, a follow up period of 30 days has been considered in this in-hospital follow-up too since it included observations from day 0 to day 30.

*Short-term follow-up time period*: included the time period after being discharged from the hospital to less than 1 year after PCI.

*Long-term follow-up time period*: included a follow up time period at 1 year or more following PCI.

### 2.4 Data extraction and quality assessment

Six authors independently reviewed the data and assessed the eligibility and methodological quality of each eligible trial or observational cohort. Information regarding study and patient characteristics, intervention strategies, and the pre-specified clinical outcomes was systematically extracted. Disagreements were discussed and resolved by consensus. The bias risk of trials was assessed with the components recommended by the Cochrane Collaboration^[6]^ and those for the observational cohorts was assessed by the Newcastle Ottawa Scale (NOS). For the trial assessment using the Cochrane Collaboration, scores were given (maximum 12 points) whereby a higher score represented a lower bias risk. For the observational cohorts, assessment using the NOS involved a total maximum score of 9 points.

### 2.5 Statistical analysis

Study selection, data collection, analysis, and reporting of the results were performed using the recommendations of the PRISMA (Preferred Reporting Items for Systematic Reviews and Meta-*Analyses*) statement.^[7]^ Heterogeneity across trials was assessed using the Cochrane *Q* statistic (*P* ≤ .05 was considered significant) and *I*^2^ statistic.^[8]^ An *I*^2^ value approaching 0% indicated low heterogeneity, and larger values indicated increased heterogeneity. A fixed effect model (*I*^2^ < 50%) or a random effect model (*I*^2^ > 50%) was used during the data analysis.

Publication bias was visually estimated by assessing funnel plots. We calculated risk ratios (RR) and 95% confidence intervals (CIs) for categorical variables. The pooled analyses were carried out with RevMan 5.3 software.

### 2.6 Ethics

This is a meta-analysis including data which were obtained from previously published studies therefore ethical approval or board review approval was not required.

## 3 Results

### 3.1 Searched outcomes

**Figure 1:**
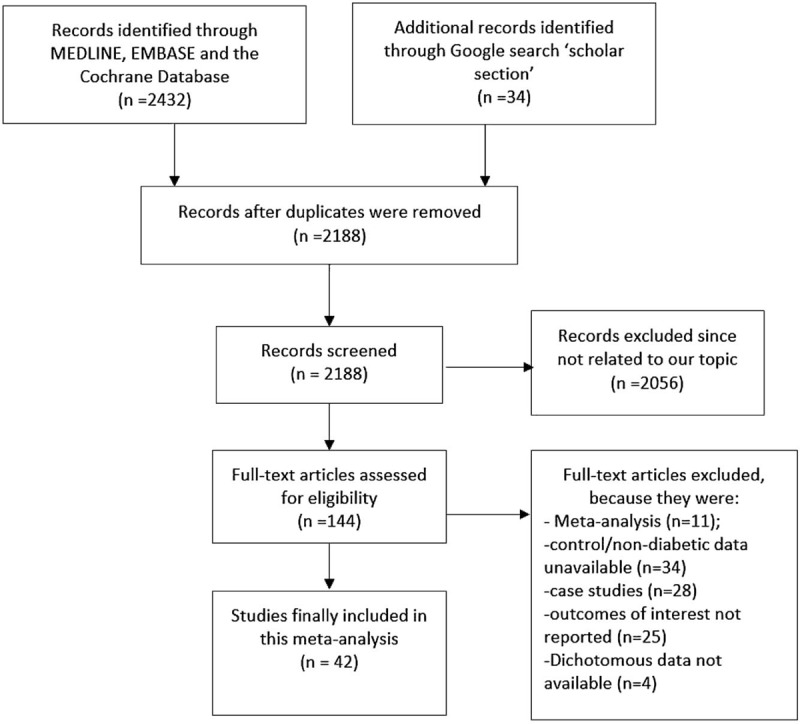
Flow diagram representing the study selection.

A total number of 2466 articles were identified from the search databases. Two thousand fifty six (2056) articles were excluded based on the titles and abstracts. Twelve (12) articles were added from references. One hundred forty four (144) full text articles were assessed for eligibility. Additional articles were excluded for the following reasons: they were meta-analyses, case studies, data for non-diabetics (control group) were not available, outcomes of interest were not reported and also discontinuous variables which were very important for the statistical analysis were not reported. The study selection has been represented in Figure 1.

A total number of 42 articles were included in this meta-analysis with a total number of 40,053 patients with T2DM and 99,721 patients without DM (T2DM + non-DM = 139,774 patients).

### 3.2 Baseline features of the studies

The baseline features of the participants have been listed in Table 1.

**Table 1:**
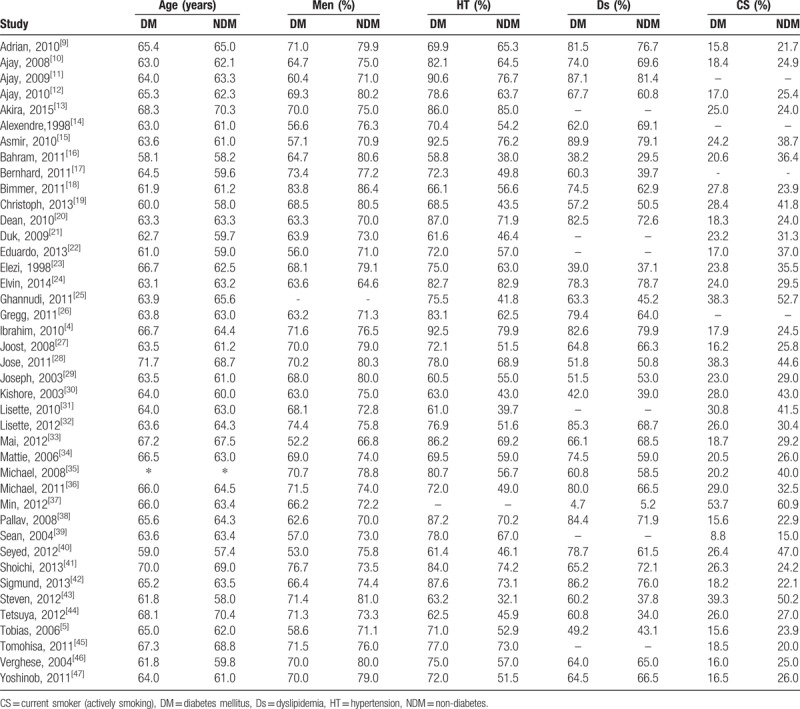
Baseline characteristics of the included studies.

∗Trial Michael 2008^[35]^ had 60.4% of patients with T2DM over the age of 65 years old while 39.6% of the patients without diabetes were over 65 years of age.

Treated hyperlipidemia was considered to be in the same category as dyslipidemia.^[12]^

Smoking and current smoking have been included in the same category.

Participants in the diabetic and non-diabetic groups were almost of similar age. However, in certain studies, patients without diabetes were younger.^[17]^ Male patients were dominant compared to female patients. Three studies consisted of more than 90% of patients suffering from hypertension.^[4,11,15]^ The percentage of patients with T2DM who smoke was less compared to patients without diabetes (exceptions: ^[13,18,41]^).

The number of participants, stent types, and the follow up time period reported in each study have been listed in Table 2.

**Table 2:**
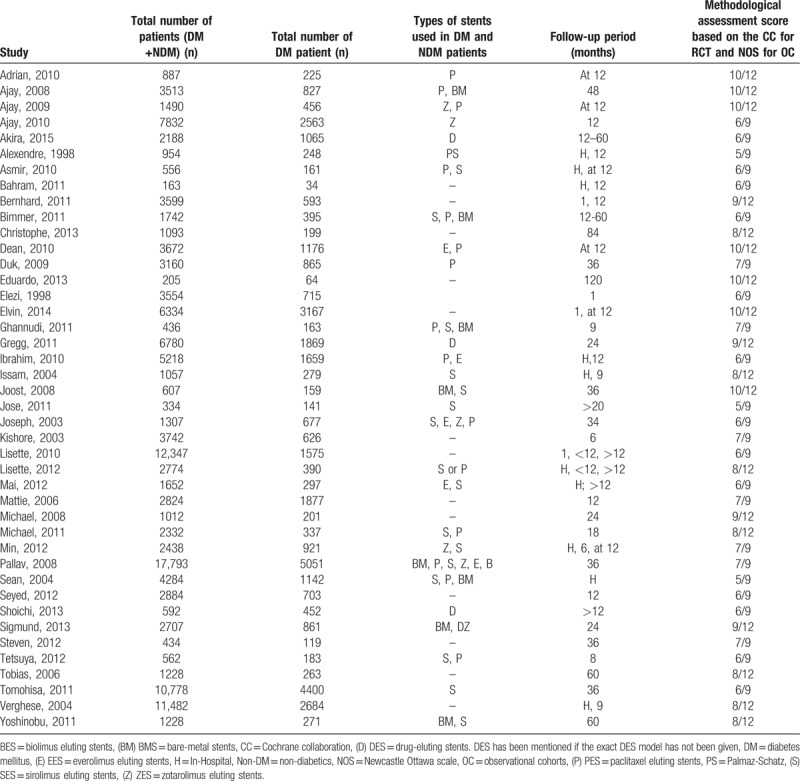
Number of patients, stents used, and the follow-up period in each study.

### 3.3 Outcomes associated with in-hospital follow up time period

**Figure 2:**
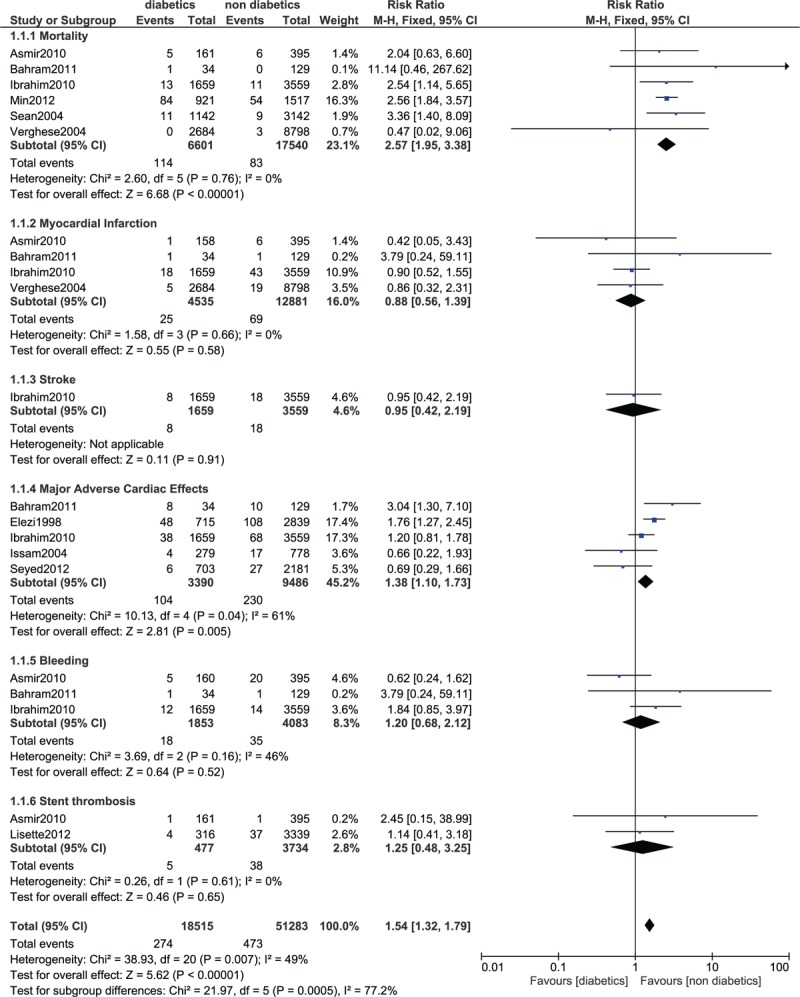
Forest plot comparing the in-hospital adverse clinical outcomes observed in patients with vs without type 2 diabetes mellitus following PCI. PCI = percutaneous coronary intervention.

In-hospital mortality was significantly higher in patients with T2DM (RR 2.57; 95% CI: 1.95–3.38; *P* < .00001). MACEs were also significantly higher in the diabetic group (RR 1.38; 95% CI: 1.10–1.73, *P* = .005). However, MI and bleeding were not significantly different during this in-hospital follow-up. Even if stent thrombosis was higher in the diabetic group, the result was not statistically significant. The result for the in-hospital follow up has been illustrated in Figure 2.

### 3.4 Outcomes associated with a short term follow up time period

**Figure 3:**
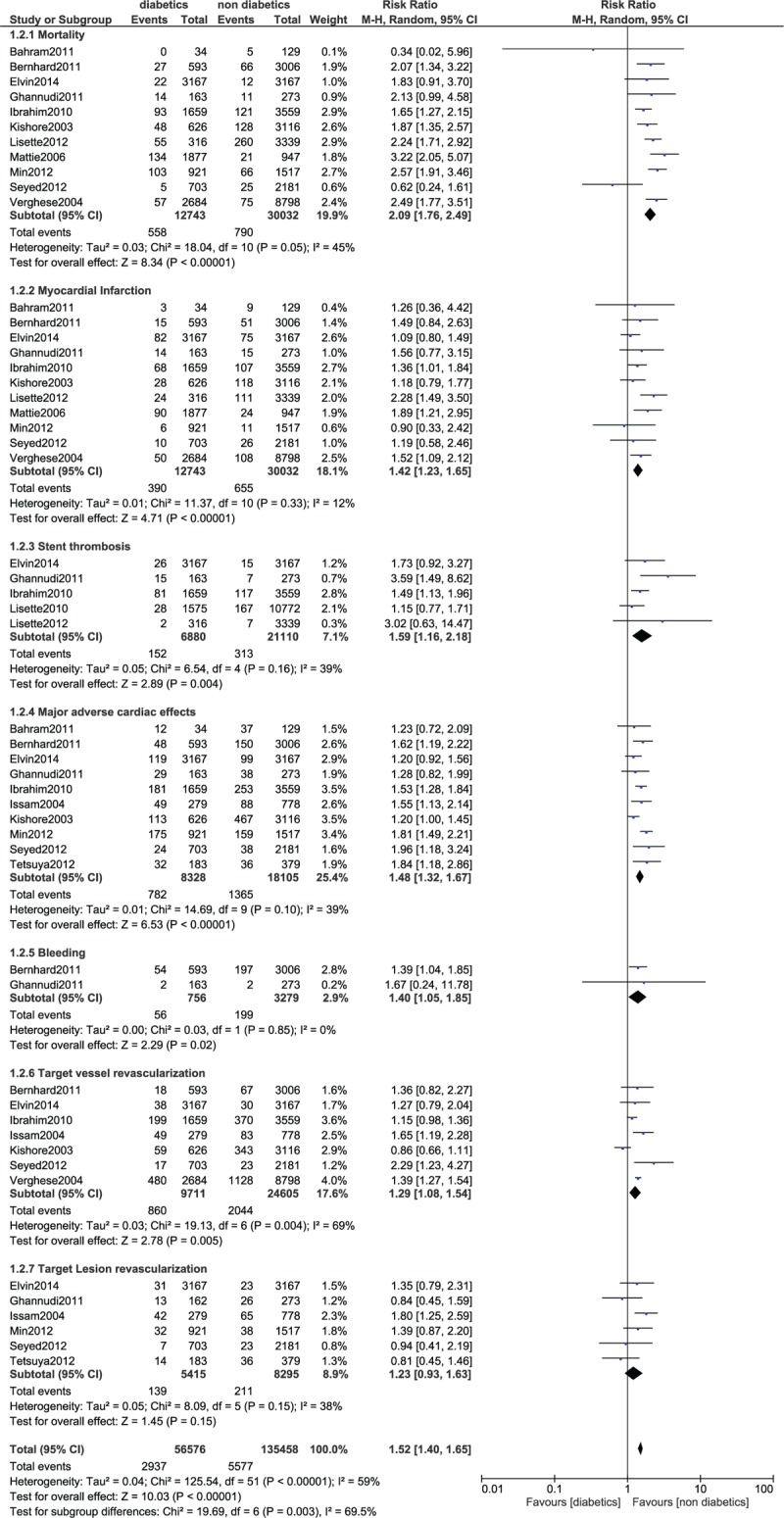
Forest plot comparing the short-term adverse clinical outcomes observed in patients with vs without type 2 diabetes mellitus following PCI. PCI = percutaneous coronary intervention.

The short term mortality was significantly higher in patients with T2DM (RR 2.09; 95% CI: 1.76–2.49, *P* < .00001). Compared to patients without diabetes mellitus (DM), MI was also significantly higher in the diabetic group (RR 1.42; 95% CI: 1.23–1.65; *P* < .00001). MACEs were also significantly higher in patients with T2DM (RR 1.48; 95% CI: 1.32–1.67; *P* < .00001) as well as bleeding (RR 1.40; 95% CI: 1.05–1.85; *P* = .02). TVR was significantly higher in the diabetic group (RR 1.29; 95% CI: 1.08–1.54; *P* = .005). In the short-term follow up period, stent thrombosis was significantly higher in the diabetic group as compared to the non-diabetic group (RR 1.59; 95% CI: 1.16–2.18; *P* = .004). The result for the short term follow up has been represented in Figure 3.

### 3.5 Outcomes at 12 months follow-up time period

**Figure 4:**
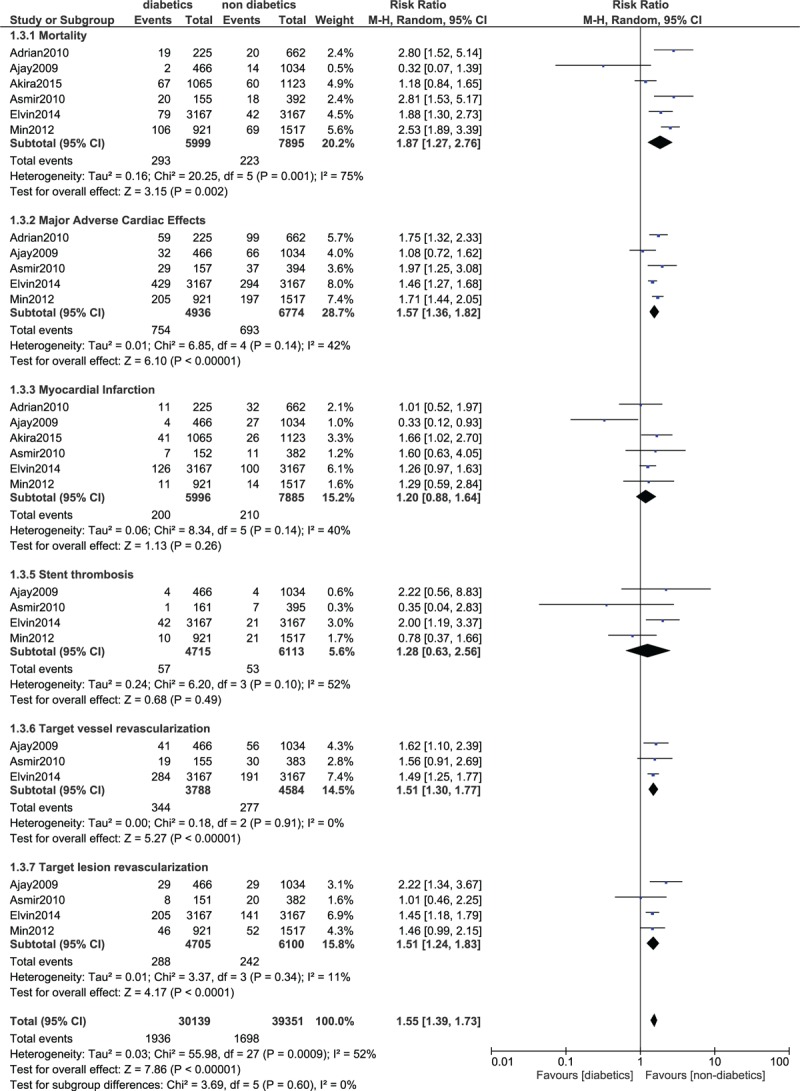
Forest plot comparing 1 year adverse clinical outcomes observed in patients with vs without type 2 diabetes mellitus following PCI. PCI = percutaneous coronary intervention.

At a follow-up time period of 1 year, mortality in patients with T2DM was significantly higher (RR 1.87; 95% CI: 1.27–2.76; *P* = .002). MACEs were also significantly higher in the diabetics (1.57; 95% CI: 1.36–1.82; *P* < .00001). TVR and TLR were also significantly higher in the diabetic group (RR 1.51; 95% CI: 1.30–1.77; *P* < .00001) and (RR 1.51; 95% CI: 1.24–1.83; *P* < .00001) respectively. However, stent thrombosis was not significantly different at 1 year follow-up. The result for the 1 year follow-up has been shown in Figure 4.

### 3.6 Outcomes associated with a long-term (>1 year) follow up time period

**Figure 5:**
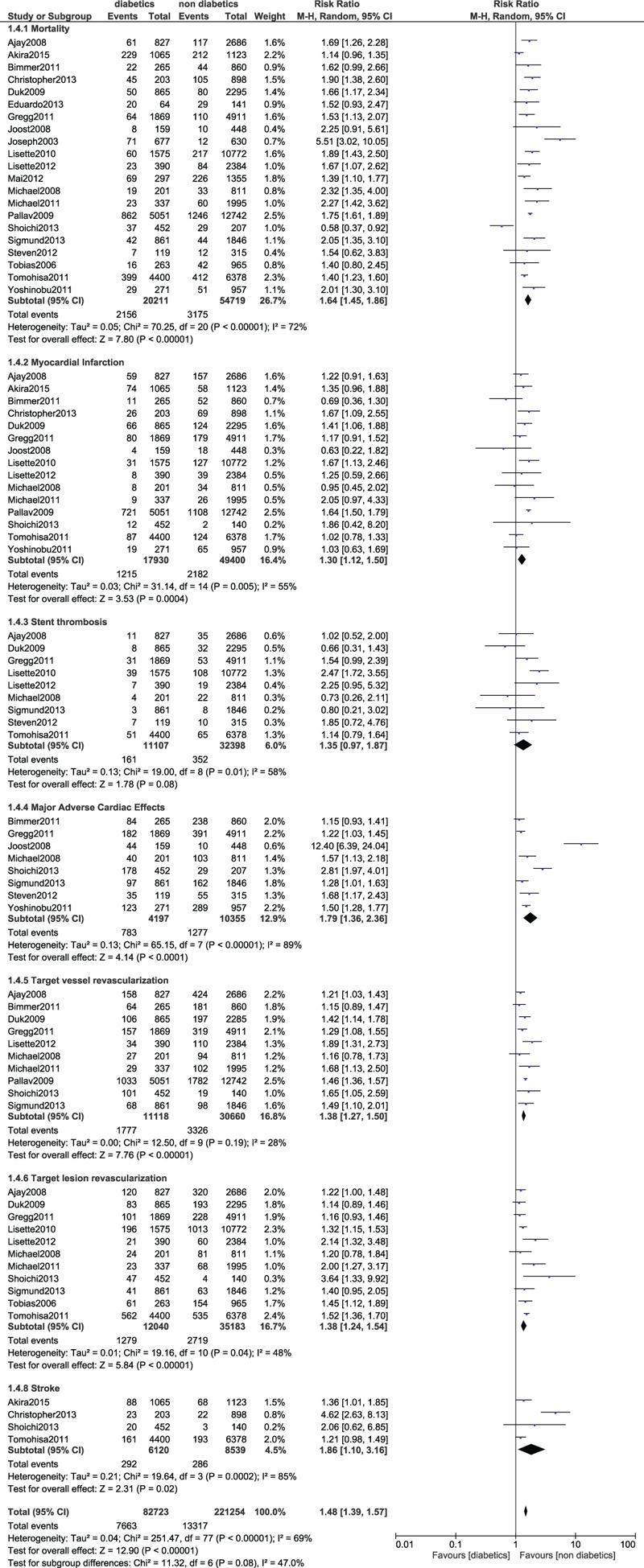
Forest plot comparing the long-term (>1 year) adverse clinical outcomes observed in patients with vs without type 2 diabetes mellitus following PCI. PCI = percutaneous coronary intervention.

Similarly, the long-term mortality was significantly higher in patients with T2DM (RR 1.64; 95% CI: 1.45–1.86, *P* < .00001). Compared to patients without diabetes, MI and MACEs were also significantly higher in the diabetic group (RR 1.30; 95% CI: 1.12–1.50; *P* = .0004) and (RR 1.79; 95% CI: 1.36–2.36; *P* < .0001) respectively. TVR and TLR were also significantly higher in the diabetic group (RR 1.38; 95% CI: 1.27–1.50; *P* < .00001) and (RR 1.38; 95% CI: 1.24–1.54; *P* < .00001), respectively. Stroke also significantly favored non-diabetics (RR 1.86; 95% CI: 1.10–3.16; *P* = .02). However, the long-term (>1 year) stent thrombosis was not significantly higher in patients with T2DM after PCI (*P* = .08). The result for the long-term (>1 year) follow up has been represented in Figure 5.

## 4 Discussion

The results of this analysis showed patients with T2DM to be more at risk of several adverse clinical outcomes after PCI whether during the in-hospital stay, short-term or long-term follow up period. Stent thrombosis has also been found to be higher in the DM patients during the short term follow-up period, however, the long-term risk was not significantly higher.

There are multiple possible explanations for the increased adverse outcomes in patients with T2DM following PCI. First of all, DM is an independent risk factor for the occurrence of cardiovascular diseases.^[2]^ DM can even aggravate serious cardiovascular impairments. Patients with T2DM are exposed to a high risk profile with associated dyslipidemia and hypertension which could to an extent, contribute to these adverse outcomes in comparison to patients without diabetes.^[37]^ Furthermore, the angiographic sub-study of the PRESTO Trial demonstrated that patients with T2DM were more likely to have new lesions on angiographic follow-up compared to non-diabetic.^[46]^

Patients with T2DM are often associated with different co-morbid conditions such as multi-vessel diseases and previous MI. Additionally, although the total number of diseased vessels (single vs double vs triple vessel disease) were comparable between diabetics and non-diabetics, patients with T2DM were exposed to more lesions treated per patient, which increased the risk of adverse events.^[9]^ Possible reasons could be vessel size and lesion length, as predictors of stent thrombosis, which might explain the predisposition of patients with T2DM to those adverse events. Other possible explanations could be the high platelet aggregations among patients with T2DM and the hypo-responsiveness to aspirin and clopidogrel which could result in stent thrombosis or even stroke.^[48–49]^ Stent thrombosis and stroke are fatal conditions which might directly result in death of the patients in several cases.

The study by Verghese et al published in 2004 also supported this current result showing that compared to non-DM patients, DM patients had a higher incidence of death, MI, and TVR during a 9 months follow-up period after PCI.^[46]^ The authors of this same study also demonstrated long-term outcomes in patients with T2DM to be clearly worse. Elvin et al showed that patients with T2DM had higher rates of death, MI and stent thrombosis compared to non-DM patients after PCI.^[24]^ Another study showed in-hospital mortality to be higher in patients with T2DM.^[30]^

The ENDEAVOR IV trial also demonstrated that patients with T2DM had higher risk of adverse outcomes compared to the control group following PCI.^[11,43]^ In the EVASTENT study or Évaluation coÛt/efficacité du stent actif au sirolimus chez les patients diabétiques et non diabétiques was a matched multicenter cohort registry, whereby 844 patients with T2DM were matched with 887 patients without DM, a total number of 45 cases of stent thrombosis were observed during the follow-up period. Of the 45 cases, 30 were definite, 8 were probable, and 7 were possible stent thrombosis. At 1 year of follow-up, stent thrombosis was 3.2% in patients with T2DM and 1.7% in the non-diabetic group.^[50]^

Nevertheless, a few studies showed results which were different from this current meta-analysis. For example, Sigmund et al showed that not a single patient with T2DM in that cohort had stent thrombosis.^[42]^ In the SORT OUT IV trial, no definite stent thrombosis was seen in patient with T2DM treated with the EES.^[32]^ Another study showed the risk of definite stent thrombosis to be lower in those patients with and without DM. The risk of definite stent thrombosis after DES vs BMS implantation also did not vary by diabetes status. However, the incidence of very late definite stent thrombosis and MI was significantly greater only in patients without diabetes treated with DESs, and only 1 patient with T2DM developed very late definite stent thrombosis.^[31]^

### 4.1 Limitations

Limitations were as followed: Several studies included patients with different co-morbidities. The comorbidity status among the patients with DM was not same in all the studies. A few studies included patients with chronic total occlusion, patients with acute coronary syndrome, patients with stable coronary artery disease, patients with single vessel and multi-vessel diseases. This might have had an impact on the results. In this current analysis, MI was mixed and analyzed, that is, Q and non-Q wave MI, fatal and non-fatal MI; and STEMI and NSTEMI were combined and analyzed as one particular subgroup. In addition, the types of stents were not taken into consideration. Patients who were implanted with bare metal stents and different drug eluting stents were analyzed together. Also, a few subgroups showed a moderate level of heterogeneity. The duration of disease, the blood sugar control status and the use of oral hypoglycemic medications or other cardiac medications were ignored. The duration length of dual antiplatelet use was also not taken into consideration and this might be considered a major limitation of this study. At last, non-atherosclerotic fatty liver disease might also have had an impact on the occurrence of cardiovascular disease in those patients and might be among the factors responsible for morbidity and mortality among the participants. However, this was not studied in this current analysis and was therefore ignored. This might also be considered as a limitation of this study.

## 5 Conclusion

According to this meta-analysis including a total number of 139,774 patients, following PCI, those patients with T2DM suffered more in-hospital, short as well as long-term adverse outcomes as reported by most of the Randomized Controlled Trials and Observational studies, compared to those patients without diabetes. Therefore, as a general message, DM is an independent factor responsible for a high risk of adverse outcomes following PCI.

## Acknowledgments

All named authors meet the International Committee of Medical Journal Editors (ICMJE) criteria for authorship for this article, take responsibility for the integrity of the work as a whole, and have given their approval for this version to be published.

## Author contributions

XZ, CZ, JF, SO, PN and ZD were responsible for the conception and design, acquisition of data, analysis and interpretation of data, drafting the initial manuscript and revising it critically for important intellectual content. XZ and CZ contributed equally to this work and wrote the final draft of this manuscript.

**Conceptualization:** Xiaojun Zhuo, Chuanzeng Zhang, Juan Feng, Shenyu Ouyang, Pei Niu, Zhaohui Dai.

**Data curation:** Xiaojun Zhuo, Chuanzeng Zhang, Juan Feng, Shenyu Ouyang, Pei Niu, Zhaohui Dai.

**Formal analysis:** Xiaojun Zhuo, Chuanzeng Zhang, Juan Feng, Shenyu Ouyang, Pei Niu, Zhaohui Dai.

**Funding acquisition:** Xiaojun Zhuo, Chuanzeng Zhang, Juan Feng, Shenyu Ouyang, Pei Niu, Zhaohui Dai.

**Investigation:** Xiaojun Zhuo, Chuanzeng Zhang, Juan Feng, Shenyu Ouyang, Pei Niu, Zhaohui Dai.

**Methodology:** Xiaojun Zhuo, Chuanzeng Zhang, Juan Feng, Shenyu Ouyang, Pei Niu, Zhaohui Dai.

**Project administration:** Xiaojun Zhuo, Chuanzeng Zhang, Juan Feng, Shenyu Ouyang, Pei Niu, Zhaohui Dai.

**Resources:** Xiaojun Zhuo, Chuanzeng Zhang, Juan Feng, Shenyu Ouyang, Pei Niu, Zhaohui Dai.

**Software:** Xiaojun Zhuo, Chuanzeng Zhang, Juan Feng, Shenyu Ouyang, Pei Niu, Zhaohui Dai.

**Supervision:** Xiaojun Zhuo, Chuanzeng Zhang, Juan Feng, Shenyu Ouyang, Pei Niu, Zhaohui Dai.

**Validation:** Xiaojun Zhuo, Chuanzeng Zhang, Juan Feng, Shenyu Ouyang, Pei Niu, Zhaohui Dai.

**Visualization:** Xiaojun Zhuo, Chuanzeng Zhang, Juan Feng, Shenyu Ouyang, Pei Niu, Zhaohui Dai.

**Writing – original draft:** Xiaojun Zhuo, Chuanzeng Zhang.

**Writing – review & editing:** Xiaojun Zhuo, Chuanzeng Zhang.
